# Complete Genome Sequence of *Desulfomicrobium* sp. Strain ZS1 from Zodletone Spring in Oklahoma, USA

**DOI:** 10.1128/mra.00145-23

**Published:** 2023-04-13

**Authors:** Sayali A. Mulay, William G. Alexander, C. Ryan Hahn, Dawn M. Klingeman, Mostafa S. Elshahed, Noha H. Youssef, Mircea Podar

**Affiliations:** a Department of Microbiology, University of Tennessee Knoxville, Knoxville, Tennessee, USA; b Biosciences Division, Oak Ridge National Laboratory, Oak Ridge, Tennessee, USA; c Department of Microbiology and Molecular Genetics, Oklahoma State University, Stillwater, Oklahoma, USA; Wellesley College

## Abstract

*Desulfomicrobium* sp. strain ZS1 is an obligate anaerobic, sulfate-reducing member of the *Desulfobacterota* from Zodletone Spring, an anoxic sulfide-rich spring in southwestern Oklahoma. Its complete genome was sequenced using a combination of Illumina and Oxford Nanopore platforms and encodes 3,364 proteins and 81 RNAs on a single chromosome.

## ANNOUNCEMENT

The anoxic sediments and air-exposed water of Zodletone Spring (Kiowa County, OK, global positioning system [GPS] coordinates 35.002444 N, 98.688167 W) host some of the most highly diverse microbial communities known, encompassing over 60 phyla and candidate phyla of *Bacteria* and *Archaea* (1). The interplay of anaerobic and aerobic sulfur-based metabolism in Zodletone may be reminiscent of physiological adaptations characteristic of microbial life during Archean Earth leading to the great oxygenation event (2). *Desulfomicrobium* sp. strain ZS1 was isolated from a Zodletone sediment sample (1) as a strict anaerobic, motile chemolithoheterotroph, reducing sulfate with lactate as electron donor. A pure culture was obtained following streaking to single colonies on DSMZ medium 63 at 25°C under 85% N_2_, 10% CO_2_, and 5% H_2_.

For genomic DNA isolation, strain ZS1 was grown in 50 mL liquid DSMZ medium 63 for 5 days at 25°C. All subsequent protocols followed manufacturers’ instructions. DNA was isolated using the Promega Wizard HMW DNA extraction kit. A short insert library was prepared using the Illumina Nextera XT DNA library preparation kit, followed by sequencing (2 × 250-nucleotide reads) on a MiSeq instrument (Illumina, Inc., San Diego, CA), yielding 1 million paired reads. Trimmomatic v0.36 (3) was used for quality-based trimming. Long-read sequencing was performed using the Oxford Nanopore ligation sequencing kit followed by sequencing on a MinION R9.4.1 device (Oxford Nanopore Technologies [ONT], Inc., Cambridge MA), yielding 1.9 Gb with an *N*_50_ of 8.2 kbp. All subsequent data analyses were performed using software defaults. Base calling and long-read polishing were performed using ONT’s Guppy and Medaka v1.5, respectively. First-pass assemblies of the long reads were generated using the Trycycler v0.5.0 pipeline (4) with Miniasm/Minpolish v0.3-r179 (5), Flye v2.9 (6), Raven v1.5.3 (7), and wtdbg2 v2.5 (8) assemblers, followed by polishing of the consensus single contig with the Illumina short reads using Polypolish v0.4.3 (9) and POLCA v4.0.5 (10) in tandem. Circularity was confirmed as part of Trycycler pipeline assembly and further verified by back mapping of the Illumina reads. The genome is 3,867,579 bp long, with an average coverage of 479× and a G+C% of 58.9.

**FIG 1:**
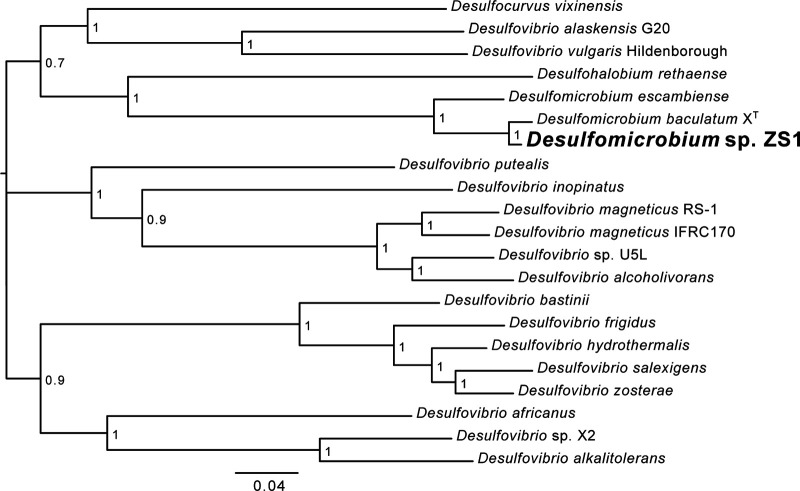
Phylogenetic tree of *Desulfomicrobium* sp. ZS1 and related *Desulfobacterota* species, based on 49 core, universal bacterial proteins. Numbers at the nodes indicate support values. The scale bar indicates estimated amino acid substitutions per site.

To predict and annotate the genes, we used the NCBI Prokaryotic Genome Annotation Pipeline (PGAP) v6.1 (11). The genome encodes 3,364 proteins and 81 RNAs, which include three rRNA operons. The gene encoding the chromosomal replication initiator protein DnaA was set as the first gene. Comparative genomic analyses were performed using software implemented in KBase (12) as follows. A phylogenetic tree constructed using SpeciesTree v2.2.0, using a set of 49 core, universal bacterial genes, placed *Desulfomicrobium* sp. ZS1 closest to Desulfomicrobium baculatum strain X^T^, the genus type species (NCBI accession number PRJNA29527) (13, 14) (Fig. 1). Whole-genome average nucleotide identity (ANI) between the two genomes, calculated using FastANI v0.1.3 (15), is 94%, suggesting that ZS1 is a species closely related to *D. baculatum. Desulfomicrobium* sp. ZS1 will facilitate studies on the evolution of microbial sulfur metabolism and adaptation to anoxic and microoxic environments.

### Data availability.

The annotated genome sequence has been deposited in GenBank under the accession number CP100351. The version described in this article is CP100351.1. The Nanopore and Illumina reads are available in the NCBI Sequence Read Archive (SRA) under the accession numbers SRR21699889 and SRR20017258, respectively.
